# Synthesis of Porous Hollow Organosilica Particles with Tunable Shell Thickness

**DOI:** 10.3390/nano12071172

**Published:** 2022-04-01

**Authors:** Mohammed A. Al-Khafaji, Anikó Gaál, Bálint Jezsó, Judith Mihály, Dorota Bartczak, Heidi Goenaga-Infante, Zoltán Varga

**Affiliations:** 1 Institute of Materials and Environmental Chemistry, Research Centre for Natural Sciences, H-1117 Budapest, Hungary; al-khafaji.mohammed@ttk.hu (M.A.A.-K.); gaal.aniko@ttk.hu (A.G.); jezso_balint@hotmail.com (B.J.); mihaly.judith@ttk.hu (J.M.); 2 Hevesy György Ph.D. School of Chemistry, Eötvös Loránd University, H-1117 Budapest, Hungary; 3 National Measurement Laboratory, LGC Limited, Teddington TW11 0LY, UK; dorota.bartczak@lgcgroup.com (D.B.); heidi.goenaga-infante@lgcgroup.com (H.G.-I.)

**Keywords:** porous hollow silica particles, organosilica, 1,2-bis(triethoxysilyl) ethane, spICP-MS

## Abstract

Porous hollow silica particles possess promising applications in many fields, ranging from drug delivery to catalysis. From the synthesis perspective, the most challenging parameters are the monodispersity of the size distribution and the thickness and porosity of the shell of the particles. This paper demonstrates a facile two-pot approach to prepare monodisperse porous-hollow silica particles with uniform spherical shape and well-tuned shell thickness. In this method, a series of porous-hollow inorganic and organic-inorganic core-shell silica particles were synthesized via hydrolysis and condensation of 1,2-bis(triethoxysilyl) ethane (BTEE) and tetraethyl orthosilicate (TEOS) in the presence of hexadecyltrimethylammonium bromide (CTAB) as a structure-directing agent on solid silica spheres as core templates. Finally, the core templates were removed via hydrothermal treatment under alkaline conditions. Transmission electron microscopy (TEM) was used to characterize the particles′ morphology and size distribution, while the changes in the chemical composition during synthesis were followed by Fourier-transform infrared spectroscopy. Single-particle inductively coupled plasma mass spectrometry (spICP-MS) was applied to assess the monodispersity of the hollow particles prepared with different reaction parameters. We found that the presence of BTEE is key to obtaining a well-defined shell structure, and the increase in the concentration of the precursor and the surfactant increases the thickness of the shell. TEM and spICP-MS measurements revealed that fused particles are also formed under suboptimal reaction parameters, causing the broadening of the size distribution, which can be preceded by using appropriate concentrations of BTEE, CTAB, and ammonia.

## 1. Introduction

Hollow silica particles (HSPs) with porous shells have recently attracted considerable attention among various nanomaterials [1,2]. The porous structure ensures the permeability of the shell for loading various cargo into the interior of the particles. Moreover, the excellent mechanical and thermal stability, easy surface functionalization, and large specific surface area place these particles in the focus of attention in many fields, including drug delivery, catalysis, and environmental chemistry [3,4,5,6]. The general approach for constructing HSPs is the so-called template-assisted method [7], which is classified into soft templating and hard templating methods [8]. In the soft templating method, the templates, such as emulsion droplets, vesicles [9,10], and micelles [11], act as soft organic cores for the porous shells growth, which are directly converted into hollow structures after core removal without additional chemical treatment [12]. Although removing soft templates after shell fabrication is relatively easy, these templates are sometimes thermodynamically unstable and quickly altered by reaction parameters, such as temperature, pH, solvent polarity, and ionic strength, producing non-uniform, hollow structures. Unlike the soft templating method, the hard templating method relies on using a solid core (e.g., inorganic particles) as a template to prepare a uniform, hollow structure [13].

Silica nanoparticles prepared by the Stӧber method are frequent hard templates due to their facile synthesis and easy size control [14,15]. Moreover, hydrofluoric acid or alkaline solutions (e.g., sodium hydroxide, sodium carbonate, or ammonium hydroxide) can dissolve silica templates easily [16,17]. The synthesis of a mesoporous silica shell on the surface of the silica template forms core-shell nanoparticles, then the selective removal of the inner core to create uniform hollow particles needs a thorough parameter optimization. Various methods have been used for the selective etching of the core of the particles, the most common being the fabrication of an organosilica shell by using 1,2-bis(triethoxysilyl) ethane (BTEE) as a precursor. In this case, the Si-C bonds make the layer more resistive for basic hydrolysis, enabling the core removal under alkaline conditions. Other strategies, such as structural difference-based selective etching (SDSE), surface-protected etching, and dissolving and reassembling strategy, have also been described. In the case of SDSE, the inner silica core is removed based on the structural difference between the solid core and the mesoporous shell with the same chemical compositions [2,18]. Using a surface-protecting compound, such as poly(vinyl pyrrolidone), inhibits the dissolution of the shell during the core removal in the case of the surface-protected etching strategy [19]. This process suffers from the formation of disordered pores with bimodal size distribution caused by anisotropic etching. Su and co-workers reported a one-pot method for preparing monodispersed hollow organosilica particles via the dissolving and reassembling process [20]. The mixture of tetraethyl orthosilicate (TEOS) and 1,4-bis(triethoxysilyl) benzene (BTSB) was used as a precursor in the presence of hexadecyltrimethylammonium bromide (CTAB) as a structure-directing agent. TEOS was first hydrolyzed and condensed with CTAB to form the porous core in this method. Then, this core is dissolved and reassembled with an organic shell to leave behind an HSP.

Despite many efforts to synthesize HSPs with controllable size and tunable shell thickness, there are still areas to explore in the field. One of the most important properties of these colloidal systems is their size distribution. The multistep preparation procedures often harm the size distribution, which is frequently overlooked because of the inappropriate use of particle characterization methods.

This work demonstrates a simple two-pot approach for preparing porous hollow inorganic and organosilica particles with uniform spherical shape and tunable shell thickness with a rigorous characterization of the final products. Monodisperse solid silica core particles with two different sizes were prepared via the Stӧber method by varying the water–ethanol–ammonia ratio. We systematically studied the influence of the concentration of precursor, CTAB, and the etching media on the morphology, shell thickness, and the size distribution of porous HSPs. This study reveals the importance of using single-particle characterization methods, such as single-particle inductively coupled plasma mass spectroscopy (spICP-MS), to identify possible fusion and aggregation during the synthesis of HSPs.

## 2. Materials and Methods

### 2.1. Materials

Tetraethyl orthosilicate (TEOS, CAS No. 78-10-4, puriss., 99% (GC)), Sigma-Aldrich, Budapest, Hungary), 1,2-bis(triethoxysilyl)ethane (BTEE) (96%; Sigma-Aldrich, St Louis, MO, USA), ethyl alcohol (CAS No. 64-17-5, 99.8%, VWR International, Budapest, Hungary), ammonium hydroxide (NH_4_OH, CAS No. 1336-21-6, 25% (*v/v*), Reanal, Budapest, Hungary), hydrochloric acid (CAS No. 7647-01-0, Reanal, Budapest, Hungary), sodium carbonate (Na_2_CO_3_, CAS No. 497-19-8, ≥99%, Reanal, Budapest, Hungary)), and hexadecyltrimethylammonium bromide (CTAB) (CAS No. 57-09-0, ≥98%, Sigma-Aldrich) were used as received without any further purification.

### 2.2. Synthesis of Solid Silica (sSiO_2_) Core Particles

Solid SiO_2_ core particles with a nominal particle size of 160 nm in diameter (sSiO_2_-160) were prepared by the Stöber method according to the procedure adopted from Chen et al. [21]. 6 mL of TEOS was rapidly added into a mixture of 74 mL ethanol, 10 mL Milli-Q water (18.2 MΩ cm^−1^), and 3.14 mL ammonia solution under constant stirring of 500 rpm at 30 °C in a 250 mL Schott Duran^®^ borosilicate glass bottle. Then the reaction was carried out for one hour before the resulting sSiO_2_-160 particles were collected by centrifugation (10 min at 5000× *g* at room temperature in Falcon™ 50 mL conical centrifuge tubes) and washed two times in Milli-Q water and two times in absolute ethanol, then dried under vacuum overnight.

Solid SiO_2_ core particles with a nominal particle size of 280 nm in diameter (sSiO_2_-280) were prepared by altering the water–ethanol–ammonia molar ratio. Six mL TEOS was rapidly added to a mixture of 20 mL Milli-Q water, 60 mL absolute ethanol, and 7.5 mL ammonia solution, with continuous stirring of 500 rpm at 30 °C. The reaction was performed for one hour, and the resulting sSiO_2_ particles were washed by centrifugation using the protocol described above.

### 2.3. Synthesis of Core-Shell Silica and Organosilica Particles (csSP and csOP)

A porous silica shell was synthesized on the surface of the previously described sSiO_2_ core particles using TEOS or the mixture of TEOS and BTEE as a precursor and CTAB as a structure-directing agent. The shell thickness was tuned by controlling the concentrations of the precursor and surfactant. First, 100 mg of sSiO_2_ core was dispersed in 20 mL ultrapure water and sonicated for 1 h in a bath sonicator (ELMASONIC S10, Elma Ultrasonic Technologies, Germany). In parallel, CTAB was dissolved in 30 mL of 1:1 aqueous ethanol solution containing ammonia in a 50 mL Schott Duran^®^ borosilicate glass bottle and stirred at 500 rpm for 30 min at room temperature. The concentrations of CTAB and ammonia for the various samples are indicated in Table 1. Next, the dispersed silica sol was added dropwise to the CTAB solution under continuous stirring. Then the mixture was heated to 30 °C, and TEOS (for csSPs) or the mixture of TEOS and BTEE (for csOPs) in different amounts indicated in Table 1, was rapidly added. The reaction was performed for 6 h, and the resulting core-shell particles were collected by centrifugation (10 min at 5000× *g* at room temperature in Falcon™ 50 mL conical centrifuge tubes) and washed with ethanol two times, and then repeated with MQ water twice.

### 2.4. Synthesis of Porous Hollow Silica and Organosilica Particles (pHSP and pHOP)

To selectively remove the inner core and leave the porous shell relatively intact, Na_2_CO_3_ was selected as the etching agent. The synthesized core-shell silica and organosilica particles (csSPs and csOPs, respectively) were resuspended in 12.5 mL of 0.6 M Na_2_CO_3_ solution and stirred (500 rpm) at 80 °C for 1 h. The resulting particles were collected by centrifugation (10 min at 5000× *g* at room temperature in Falcon™ 15 mL conical centrifuge tubes), washed with MQ water two times and then repeated two times with ethanol. Finally, the synthesized hollow particles were resuspended in 10 mL of ethanol solution containing 10% concentrated HCl and stirred at 60 °C for 3 h to remove CTAB. The last step was repeated three times to ensure complete removal of the CTAB. The final products were resuspended in 20 mL MQ water. Different particles are named as porous hollow silica particle-160/280_xyz (pHSP-160/280-xyz) and as porous hollow organosilica particle-160/280-xyz (pHOP-160/280-xyz) based on the type of the used precursor, where the first three digits refer to the nominal diameter of the sSiO_2_ core (i.e., 160 nm or 280 nm), while x, y, and z refer to the amount of precursor (TEOS or BTEE), ammonia, and surfactant (CTAB), as shown in Table 1.

### 2.5. Characterization

#### 2.5.1. Dynamic Light Scattering (DLS)

DLS measurements were performed on a W130i instrument (AvidNano, High Wycombe, UK) using low-volume disposable cuvettes (UVette, Eppendorf Austria GmbH, Wien, Austria). Samples were diluted 10-fold prior to measurements with MQ water. Z-Average diameter (harmonic intensity averaged particle diameter) and polydispersity index (PDI) were used to report DLS results. 

#### 2.5.2. Transmission Electron Microscopy (TEM)

For TEM investigations, 2 μL of the particle solution was pipetted onto a formvar-coated 400 mesh copper grid and incubated for 2 min, followed by removing the excess liquid by blotting with a filter paper. Images were obtained on a JEOL TEM 1011 (JEOL Ltd., Tokyo, Japan) transmission electron microscope operating at 80 kV and equipped with a Morada TEM 11 MPixel camera (Olympus, Tokyo, Japan). The particle size distributions (PSDs) were obtained by counting more than 100 particles using ImageJ (IJ 1.46r, National Institutes of Health, Bethesda, MD, USA).

#### 2.5.3. Fourier-Transform Infrared Spectroscopy (FT-IR)

Attenuated total reflection FT-IR spectroscopy using a Varian Scimitar 2000 FTIR spectrometer (Varian Inc., Palo Alto, CA, USA) equipped with an MCT (mercury–cadmium–telluride) detector and a single reflection ATR unit (Golden Gate, Specac Limited, Orpington, UK) with a diamond ATR element was used to investigate the compositional and structural properties of the prepared samples. For the measurements, 2 μL undiluted sample was air-dried onto the ATR crystal and 128 spectral scans were recorded.

#### 2.5.4. Single-Particle Inductively Coupled Plasma Mass Spectrometry (spICP-MS)

spICP-MS analysis was performed using an Agilent 8900 ICP-MS/MS (Agilent Technologies, Tokyo, Japan) instrument with a conventional MicroMist nebulizer and cooled (2 °C) spray chamber. The instrument was equipped with the MassHunter 4.3 (version: G72dC C.01.03) software and microsecond detection capability, enabling analysis in the sp mode. Measurements in fast transient analysis (TRA) mode were carried out using a dwell time of 100 μs per point, with no settling time between the measurements and using the newly developed Single Particle Application Module of the ICP-MS MassHunter software (G5714A). Total acquisition time was fixed at 60 s for all analyses. For Si detection, *m/z* 28 was measured using single quadrupole mode and with hydrogen cell gas (2.0 mL min^−1^) used to eliminate potential polyatomic interferences, mostly related to the presence of polyatomic ions, such as CO, N_2_, and N_2_H. After verifying the instrument’s performance with 1 µg/L Agilent Tuning solution, the ICPMS conditions were optimized to obtain maximum ^28^Si sensitivity with a minimum background contribution using 10 µg/L ionic silicon solution (Romil). For the purpose of this work, spICP-MS was only used to monitor the frequency distribution of the Si signal intensity, since this is particularly useful to monitor potential NP aggregation caused by the synthesis process. All samples were diluted in 1 µM sodium dodecyl sulfate ‘SDS’ (Merck) aqueous solution, pH 7.4, prior to the analysis.

#### 2.5.5. Small-Angle X-ray Scattering (SAXS)

SAXS was used to characterize the porous structure of the prepared hollow organosilica particles. Measurements were performed using an in-house built SAXS pinhole camera (CREDO) equipped with a GeniX^3D^ Cu ULD integrated beam delivery system (Xenocs SA, Sassenage, France) and a Pilatus-300k CMOS hybrid pixel detector (Dectris Ltd., Baden, Switzerland) [22]. Samples were filled into thin-walled (~0.01 mm) borosilicate capillaries with an approximately 1.3 mm outer diameter (Hilgenberg GmbH, Malsfeld, Germany). The two-dimensional scattering patterns were azimuthally averaged yielding radial scattering curves.

## 3. Results and Discussion

### 3.1. Size and Morphology of Solid Silica (sSiO_2_) Core Particles

**Figure 1:**
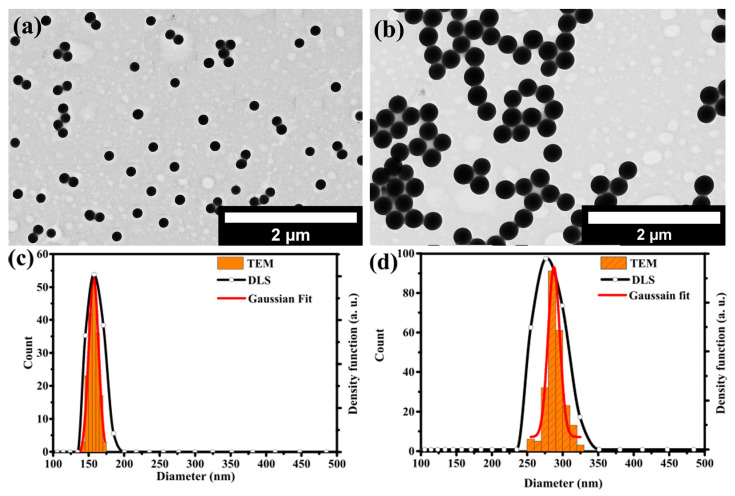
TEM images of sSiO_2_ spherical particles with (**a**) 160 nm and (**b**) 280 nm nominal particle diameter prepared via Stӧber method. (**c**,**d**) Particle size distributions as determined by TEM and DLS for both sSiO_2_ core particles.

Figure 1a,b shows the TEM images of sSiO_2_-160 and sSiO_2_-280 core particles, respectively. Both sSiO_2_ particles exhibit uniform spherical shape with no sign of aggregation or fusion of the particles. DLS measurements confirmed the absence of aggregates. The size distributions determined from the TEM images and DLS measurements are shown in Figure 1c,d for the solid SiO_2_-160 and SiO_2_-280 core particles, respectively. The mean values and standard deviations of the Gaussian functions fitted to the size distributions determined from the TEM images are shown in Table 2, together with the hydrodynamic diameters and PDIs of the particles determined by DLS.

### 3.2. Size and Morphology of Core-Shell Silica and Organosilica Particles (csSP and csOP)

**Figure 2:**
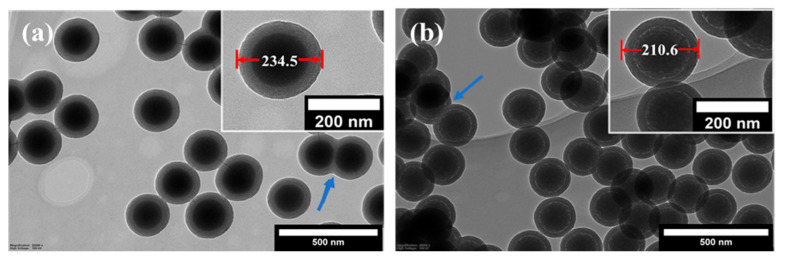
TEM images of core-shell (**a**) silica (csSP-160-103) and (**b**) organosilica (csOP-160-203) particles prepared by using TEOS and BTEE precursors, respectively.

The structural and morphological properties of the core-shell particles prepared with TEOS and BTEE precursors are illustrated using the samples csSP-160-103 and csOP-160-203, respectively. The formation of lower electron density layers on the surface of sSiO_2_-160 particles can be identified in the TEM images shown in Figure 2. The outer diameter of the core-shell particles increased from 160 nm to about 235 nm and 210 nm after shell formation for the csSP-160-103 and the csOP-160-203 samples, respectively. On the other hand, the shell formation occasionally caused the fusion of two or more particles, as highlighted in the TEM images with blue arrows. This phenomenon has led to a broadening of the particle size distributions, which will be discussed in a later section on the effect of various synthesis parameters on the structure of the final hollow particles’ inner core, which is presented in the next section.

Another structural feature of the core-shell particles prepared with BTEE is a low electron density ring on the core-shell boundary (Figure 2b inset). In parallel, the core size decreased from about 160 nm to 130 nm for the csOP-160-203 sample, which is attributed to the partial hydrolysis of the solid core. Due to the steric hindrance caused by the hydrophobicity of ethylene bridges in BTEE, the nucleophilic attack of hydroxyl ions at the silanol groups on the core surface becomes more favorable when BTEE is used as the precursor. On the contrary, the nucleophilic attack of hydroxyl ions becomes less favorable when the hydrophilic TEOS is used as the precursor. These processes affect the dissolution of the inner core, which is presented in the next section.

### 3.3. Size and Morphology of Porous Hollow Silica and Organosilica Particles (pHSP and pHOP)

#### 3.3.1. Influence of the Composition and Shell Thickness on Core Removal

**Figure 3:**
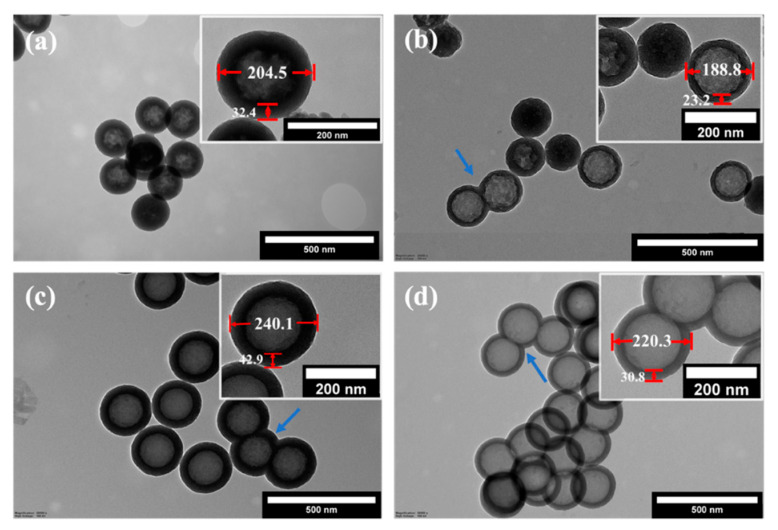
TEM images of pHSP-160-103 (**a**), pHSP-160-203 (**b**), pHOP-160-103 (**c**), and pHOP-160-203 (**d**).

The final step of preparing hollow particles is removing the inner core via etching in a basic medium. This section demonstrates the effect of the shell type and the precursor′s concentration on the morphology of the resulting hollow particles. In all cases, etching was performed for one hour at 80 °C using 0.6 M Na_2_CO_3_ solution. The hydroxyl ions diffuse inside the shell through the porous channels and dissolve the inner solid silica core to form water-soluble sodium silicate during etching. Figure 3 shows the TEM images of the resulting hollow particles prepared using TEOS (pHSP-160-103, pHSP-160-203) or BTEE (pHOP-160-103, pHOP-160-203) as the precursor for the shell with two different shell thicknesses created by varying the concentration of precursors.

Table 3 summarizes the average diameters and shell thicknesses for these four samples based on the analysis of the TEM pictures. There is an apparent difference in the morphology of the hollow particles with shells made using TEOS (pHSPs, Figure 3a,b) or BTEE (pHOPs, Figure 3c,d). pHOPs show regular morphology with complete etching of the core. pHSPs exhibit more irregular shells than pHOPs, and incomplete etching of the core can be found for 17% and 65% of the particles (estimated based on more than 100 particles from TEM images) for pHSP-160-103 and pHSP-160-203, respectively. The appearance of incompletely etched particles is more frequent for pHSP-160-103, which has a thicker shell than pHSP-160-203 due to the higher concentration of the precursor. Similarly, decreased shell thickness and the outer diameter can be observed having decreased BTEE concentration for pHOPs. The barrier layer in the case of the core-shell particles prepared with BTEE, discussed in the previous section, can serve as an explanation for these observations, as well as the fact that Si-C bonds in the shell framework of the pHOPs exhibit more chemical resistance against basic etching than that of Si-O bonds in pHSPs. Fused particles observed for csSP and csOP samples can also be observed for pHSPs and pHOPs.

#### 3.3.2. Influence of CTAB on Size and Shell Thickness of pHOPs

**Figure 4:**
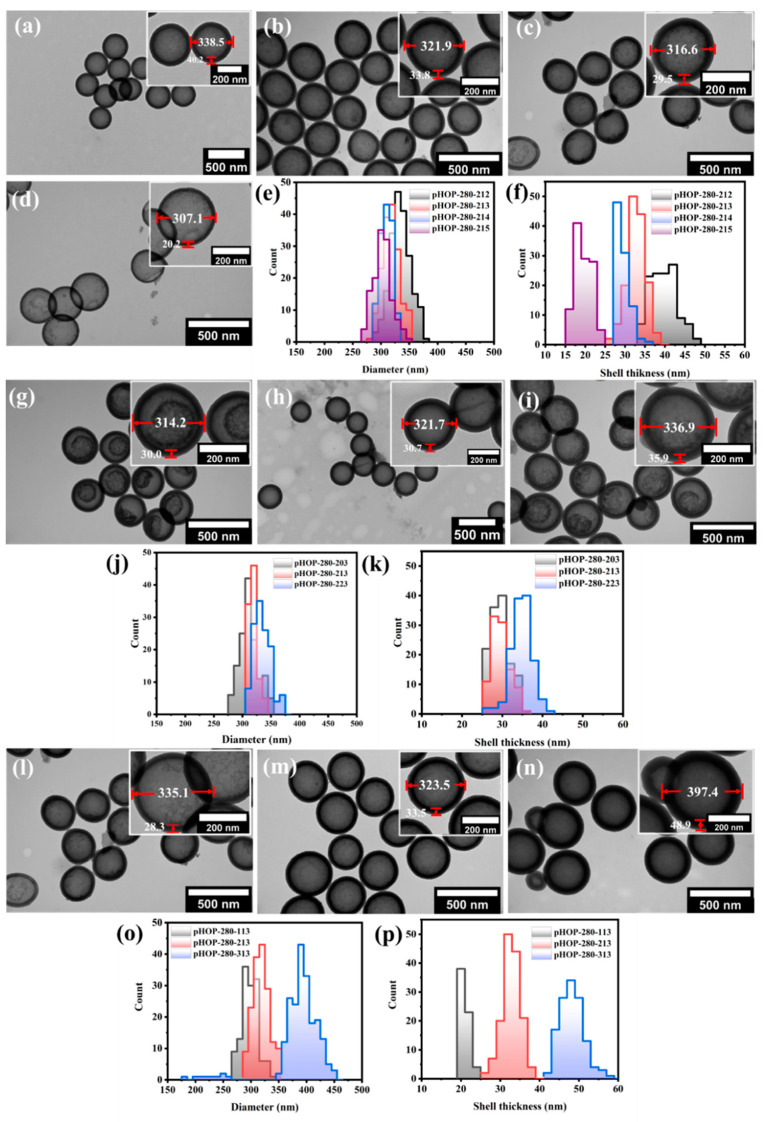
Effect of the concentration of CTAB, ammonia, and BTEE on the structure of pHOPs. TEM images of pHOPs prepared at CTAB concentrations of 5.6, 3.6, 1.8, and 0.3 mg/mL (pHOP-280-212 (**a**), pHOP-280-213 (**b**), pHOP-280-214 (**c**), and pHOP-280-215 (**d**), respectively), and the calculated outer diameter (**e**) and thickness (**f**) distributions. TEM images of pHOPs prepared at 10.2, 22.4, and 33 uL/mL ammonia concentrations (pHOP-280-203 (**g**), pHOP-280-213 (**h**) and pHOP-280-223 (**i**)), and the calculated outer diameter (**j**) and thickness (**k**) distributions. TEM images of pHOPs prepared with 25 μL, 50 μL, and 100 μL BTEE (pHOP-280-113 (**l**), pHOP-280-213 (**m**) and pHOP-280-313 (**n**)), and the calculated outer diameter (**o**) and thickness (**p**) distributions.

As demonstrated in the previous section, the use of BTEE for the preparation of the shell results in more regular and homogeneously etched hollow particles. Next, we investigated the effect of the surfactant (CTAB) concentration on the shell thickness and average size of the resulting pHOPs. Samples with decreasing CTAB concentrations of 5.6, 3.6, 1.8, and 0.3 mg/mL were prepared using the sSiO_2_-280 as core particles (pHOP-280-212, pHOP-280-213, pHOP-280-214, and pHOP-280-215, respectively). As the TEM images show in Figure 4a–f, the particles′ outer diameter and the average shell thickness decrease with decreasing CTAB concentration. The analysis of the TEM images of the samples shows that the average outer diameter of the particles decreased from 339, 322, and 317 to 307 nm and the shell thickness from 40, 34, and 30 to 20 nm with decreasing CTAB concentration.

#### 3.3.3. Influence of the Ammonia Concentration on the Etching of the Core-Shell Particles

Figure 4g–i shows the TEM images of pHOPs prepared at different ammonia concentrations. Three different ammonia concentrations of 10.2 μL/mL, 22.4 μL/mL, and 33 μL/mL (pHOP-280-203, pHOP-280-213, and pHOP-280-223, respectively) were used. The average size of pHOPs increased from 314.2 to 321.9 and 336.9 nm, with a slight increase in shell thinness from 30.0 to 33.8 and 35.9 nm by increasing the ammonia concentration (Figure 4j,k), respectively). Although all samples were etched under the same conditions (0.6 M Na_2_CO_3_ at 80 °C for 1 h), some of these samples showed incomplete core removal (Figure 4g,i). This phenomenon can be attributed to the difference between the rate of hydrolysis and condensations of the precursors. The 22.4 μL/mL NH_3_ concentration was chosen to be the optimal condition for preparing homogeneously etched pHOPs.

#### 3.3.4. Influence of the BTEE Concentration on Shell Thickness of pHOPs

The average size and shell thickness of pHOPs can be precisely tuned by changing the precursor′s concentration. Figure 4l–n shows TEM images of pHOPs prepared at BTEE amounts of 25 μL, 50 μL, and 100 μL (pHOP-280-113, pHOP-280-213, and pHOP-280-313, respectively). By increasing the amount of BTEE from 25 μL to 50 μL and 100 μL, the average diameter of the particles increased from 302.9 nm to 323.5 nm and 397.4 nm, respectively (Figure 4o), and the shell thickness increased from 21 nm to 33.8 nm and 48.9 nm (Figure 4p), respectively. On the other hand, smaller by-product particles appear at the highest BTEE concentration. This observation could be attributed to the hydrolysis of BTEE at large concentrations, which is much faster than the rate of condensation. Thus, the self-assembly of BTEE becomes preferable over the co-assembly on the surface of the silica core.

### 3.4. Characterization of Solid, Core-Shell, and Porous Hollow Silica Particles with FT-IR

**Figure 5:**
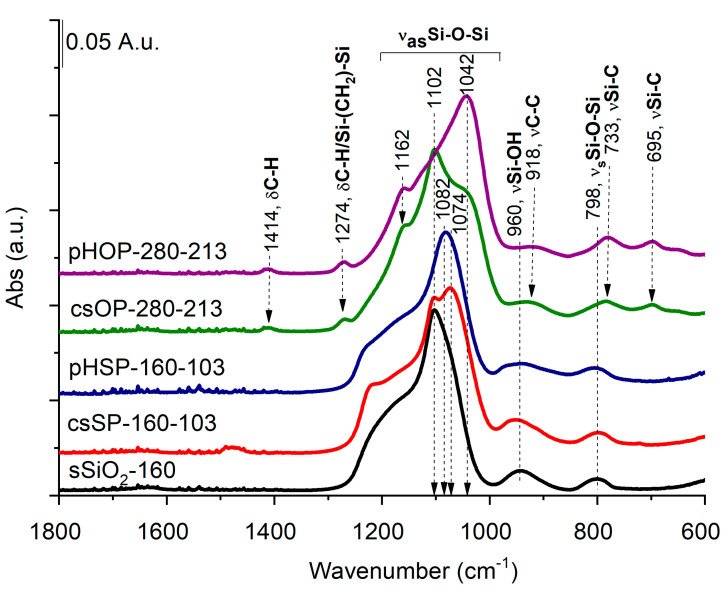
FT-IR analysis of solid (sSiO_2_), core-shell (csSP and csOP), and porous hollow silica particles (pHSP and pHOP).

Structural differences between solid (sSiO_2_), core-shell (csSP and csOP), and porous hollow silica particles (pHSP and pHOP) at the molecular level were studied by FT-IR spectroscopy. The FT-IR spectrum of the sSiO_2_ core (Figure 5) is dominated by the strong band at around 1102 cm^−1^, owing to Si-O-Si asymmetric stretching vibrations (ν_as_Si -O-Si) with a shoulder around 1192 cm^−1^, attributing to the splitting of longitudinal and transverse optical stretching motions [23]. The smaller bands at 798 and 960 cm^−1^ can be attributed to Si-O-Si symmetric and Si-OH stretching [24].

While the band with a maximum at 1102 cm^−1^ can be assigned to the nonporous sSiO_2_ core with a regular tetrahedron SiO_4_ structure, a new band at 1074 cm^−1^ appears in the csSP spectrum (Figure 5). This lower frequency band belongs to the deformed SiO_4_ tetrahedron structure in csSP, according to Grill et al. [25], which suggests that the solid silica core and porous silica shell have different structures. After core removal (pHSP), the spectrum shows a remarkable decrease in the band at 1102 cm^−1^, assigned previously to asymmetrical stretching vibrations of Si-O-Si of the sSiO_2_ core. Simultaneously, the band at 1082 cm^−1^ increased noticeably. These spectral changes indicate that the sSiO_2_ cores were removed, forming a porous silica shell in line with the TEM pictures.

Regarding the FT-IR spectrum of the core-shell organosilica particles (csOP), again, new shoulders appear in the asymmetric Si-O-Si stretching region at 1162 and 1042 cm^−1^. Several new bands also appear that can be assigned to C-H deformations (at 1414 and 1274 cm^−1^) from Si-CH_2_-CH_2_-Si moieties, C-C stretching at 918 cm^−1^, and Si-C stretching bands at 733 and 695 cm^−1^, further confirming the formation of BTEE shell. Core removal (pHOP) leads to the suppression of the asymmetric Si-O-Si stretching band 1102 cm^−1^, assigned to sSiO_2_, while the bands related to the organosilica shell remain intact. Similar to pHSP, the shift of the main Si-O-Si asymmetric stretching band towards the lower wavenumber (at 1042 cm^−1^) suggests forming of a porous silica structure. All these results agree with that obtained by TEM measurements and affirm the formation of porous hollow silica (pHSP) and organosilica (pHOP) particles.

### 3.5. Monitoring the Frequency of the Si Signal Intensity with spICP-MS

In spICP-MS, highly diluted nanoparticle suspension is introduced continuously into an ICP-MS system, with the intent that only one particle at a time arrives at the detector, which is set to acquire data with a high time resolution (i.e., dwell time). Following the nebulization, a fraction of the nanoparticles enters the plasma where they are atomized, and the individual atoms are ionized. Every atomized particle results in a cloud of ions, which is then sampled by the mass spectrometer, which can be tuned to measure any specific element. In the work presented here, spICP-MS was used as a confirmatory technique for qualitative monitoring of the frequency of Si signal distribution of pHOP samples produced under different conditions.

**Figure 6:**
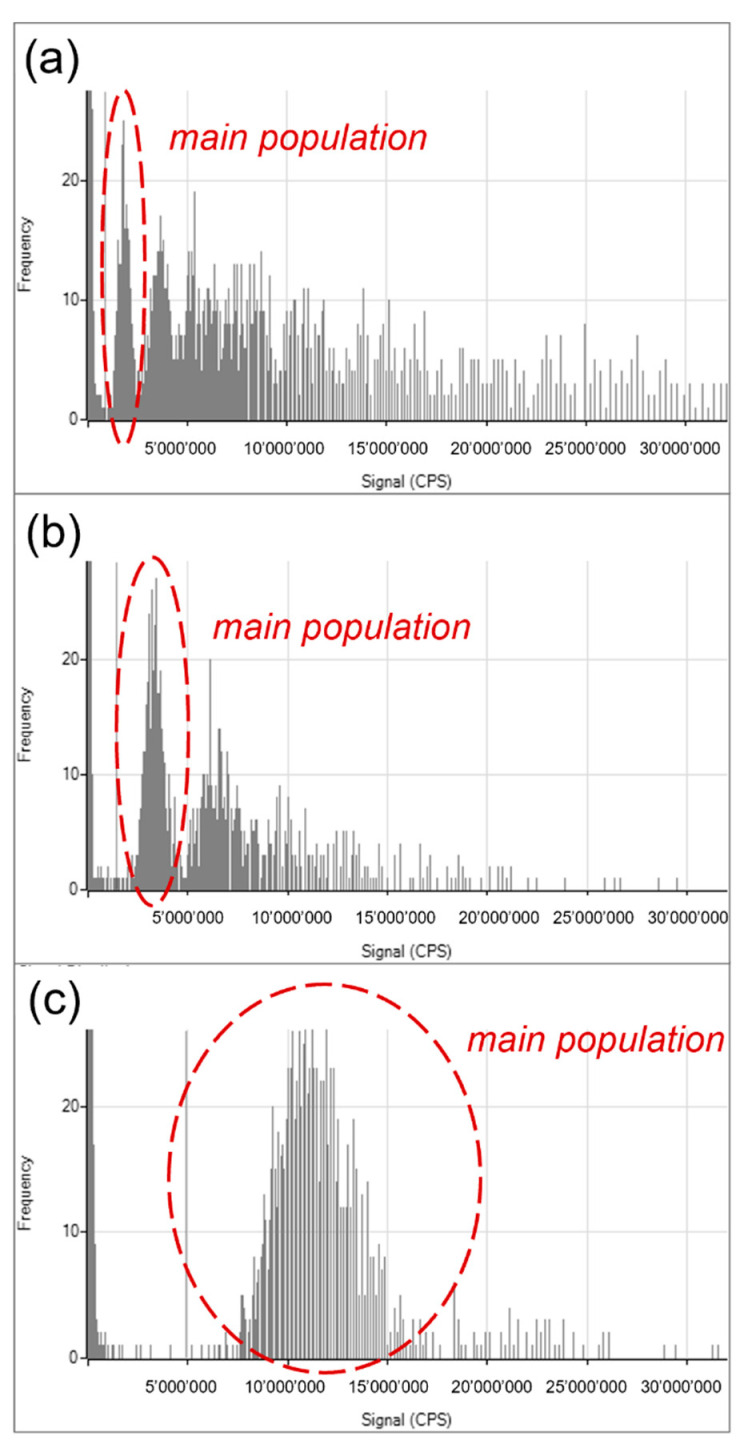
Si signal distribution of pHOPs by spICP-MS. pHOP-160-103 (**a**), pHOP-160-203 (**b**) and pHOP-280-213 (**c**).

Since the TEM images of the pHOP samples indicated the presence of fused particles, spICP-MS measurements on selected samples were performed to assess the Si signal distribution of the hollow particles. It is noted that light scattering methods, such as DLS, can give misleading results for particles with multimodal or polydisperse size distributions, as discussed in our previous work [26]. Therefore, it is essential to use a method based on single-particle detection, such as spICP-MS, to monitor the polydispersity of pHOPs. Figure 6 shows the Si signal distribution of the pHOP-160-103 (Figure 6a), pHOP-160-203 (Figure 6b), and pHOP-280-213 (Figure 6c) samples.

It can be seen from the distributions of pHOP-160-103 and pHOP-160-203 that multiple populations of particles are present in these samples besides the main population. The pHOP-160-103 sample shows the most polydisperse distribution, where the fused and aggregated particles outnumber the main population of single particles. The pHOP-160-203 sample resembles a bimodal distribution, but larger aggregates can also be found. In contrast, the distribution of the pHOP-280-213 sample is monomodal, in agreement and supporting the conclusion from the TEM analysis. 

### 3.6. Characterization of the Porosity of pHOPs with SAXS and Colloidal Stability with DLS

CTAB, as a structure-directing agent, leads to the formation of a mesoporous silica structure, which is widely established in the literature. This porous structure is indispensable for the final etching process, as hydroxyl ions must diffuse through the shell to dissolve the core template. For the quantitative characterization of the porosity of the prepared pHOPs, we used SAXS. Figure S1 shows the scattering curve of the pHOP-0213 sample, which exhibits a well-defined peak corresponding to the (100) reflection of the hexagonal lattice of the mesopores. The peak position at q = 1.608 nm^−1^ corresponds to a real-space distance (d = 2π/q) of 3.9075 nm, which gives an inter-channel distance of $a=2d/\sqrt{3}=4.512$ nm. This value agrees well with literature data and proves that the prepared pHOPs belong to the MSN-41 type of mesoporous silica particles [27,28].

The stability of the pHOP-0213 sample was assessed with DLS. The change in the average hydrodynamic diameter is below 5% for 3 months, while the PDI of the distribution remains practically unchanged, indicating sufficient colloidal stability.

## 4. Conclusions

In the present study, a facile two-pot strategy was applied to prepare sub-micrometer sized porous-hollow particles with controllable size and shell thickness. pHSPs prepared using only TEOS for the shells showed a more irregular structure than pHOPs, which were prepared using the mixture of TEOS and BTEE. This observation can be explained by the partial hydrolysis of the surface of the core particles when BTEE is used to grow the porous layer, which promotes the formation of regular hollow particles.

The effect of various reaction parameters on the size and shell thickness of pHOPs was also investigated. By changing the concentration of the precursor (BTEE), surfactant, and ammonia, it was found that the shell thickness of pHOPs increases with increased concentrations of these components. These reaction parameters also affected the size distribution and morphology of the final particles. At high BTEE concentration, by-product particles are also formed with smaller sizes, while an incomplete etching of the inner core was observed if too low or too high ammonia concentration was used.

FT-IR spectroscopic analysis was used to follow the formation of the porous hollow particles and to affirm the complete etching of the inner core. TEM investigations confirmed the regular shell structure of pHOPs. On the other hand, the appearance of fused particles was observed for most of the samples. spICP-MS measurements were performed to monitor the Si signal distributions of the particles, supporting the dispersity results from TEM. It was found that optimal reaction parameters corresponding to the pHOP-280-213 sample results in a monodisperse distribution.

## Figures and Tables

**Table 1:** List of investigated samples and their relevant experimental parameters.

Sample Name (Final Hollow Particle)	Sample Name (Core-Shell Particle)	sSiO_2_ Core Size (nm) ^1^	TEOS (μL)	BTEE (μL)	NH_3_ (μL/mL)	CTAB (mg/mL)
pHSP-160-103	csSP-160-103	160	100	0	10.2	3.6
pHSP-160-203	csSP-160-203	160	50	0	10.2	3.6
pHOP-160-103	csOP-160-103	160	10	100	10.2	3.6
pHOP-160-203	csOP-160-203	160	10	50	10.2	3.6
pHOP-280-212	csOP-280-212	280	10	50	22.4	4.6
pHOP-280-213	csOP-280-213	280	10	50	22.4	3.6
pHOP-280-214	csOP-280-214	280	10	50	22.4	1.8
pHOP-280-215	csOP-280-215	280	10	50	22.4	0.3
pHOP-280-203	csOP-280-203	280	10	50	10.2	3.6
pHOP-280-223	csOP-280-223	280	10	50	33.6	3.6
pHOP-280-113	csOP-280-113	280	10	25	22.4	3.6
pHOP-280-313	csOP-280-313	280	10	100	22.4	3.6

^1^ Nominal values.

**Table 2:** Hydrodynamic diameter (Dh) and polydispersity index (PDI) measured by DLS and mean particle size and standard deviation (SD) values from TEM for sSiO_2_ core particles prepared at different mole ratios of TEOS: EtOH: H_2_O: NH_3_.

Sample	TEOS:EtOH:H_2_O:NH_3_ Mole Ratio	DLS		TEM	
		Dh (nm)	PDI	D (nm)	SD (nm)
sSiO_2_-160	1:47:20:1.2	166.1	0.05	156.8	16.4
sSiO_2_-280	1:38:41:3	292.3	0.08	279.3	37.7

**Table 3:** Structural parameters of the hollow particles with shells made using TEOS (pHSPs) or BTEE (pHOPs) based on TEM.

Sample	Mean Outer Diameter (nm)	Mean Shell Thickness (nm)
pHSP-160-103	204.5	32.4
pHSP-160-203	188.8	23.4
pHOP-160-103	240.1	42.9
pHOP-160-203	220.8	30.8

## Data Availability

The data is available on reasonable request from the corresponding author.

## Supplementary Materials

The following are available online at https://www.mdpi.com/article/10.3390/nano12071172/s1, Figure S1: SAXS curve of the pHOP-0213 sample.
