# Supplementary material for: Synthesis of Porous Hollow Organosilica Particles with Tunable Shell Thickness
Source: Nanomaterials (Basel). 2022 Apr 1;12(7):1172. doi: 10.3390/nano12071172 (PMC9000660; doi:10.3390/nano12071172)
Supplement: Supplementary file 1 [file nanomaterials-12-01172-s001.zip › nanomaterials-1653866-supplementary.pdf]

nanomaterials

Supplementary Materials

Synthesis of Porous Hollow Organosilica Particles with Tunable Shell Thickness

Mohammed A. Al-Khafaji, Anikó Gaál, Bálint Jezsó, Judith Mihály, Dorota Bartczak, Heidi Goenaga-Infante and Zoltán Varga

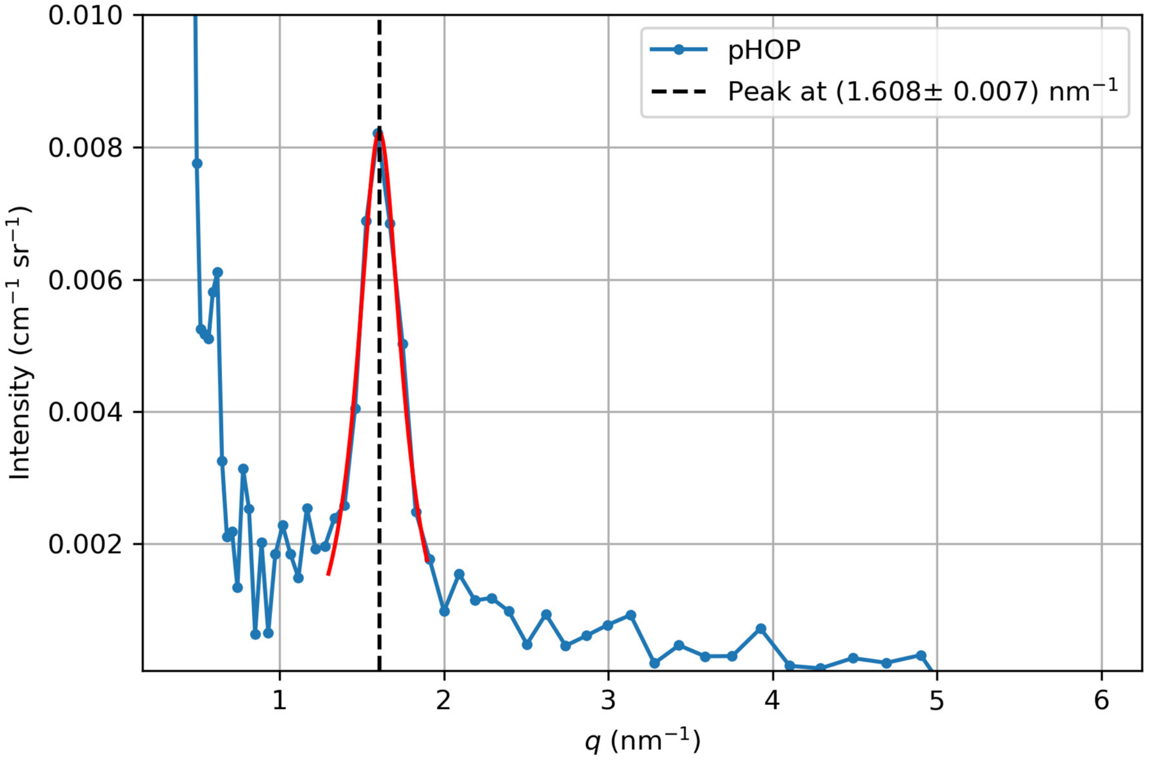

Figure S1. SAXS curve of pHOP-0213 sample. The peak at q = 1.608 nm^{-1} corresponds to the (100) reflection of the hexagonal lattice of the mesopores.
